# In vitro assessment of Lipiodol-targeted radiotherapy for liver and colorectal cancer cell lines

**DOI:** 10.1038/sj.bjc.6690266

**Published:** 1999-04

**Authors:** R A M Al-Mufti, R B Pedley, D Marshall, R H J Begent, A Hilson, M C Winslet, K E F Hobbs

**Affiliations:** Departments of SurgeryClinical Oncology, Nuclear Medicine, Royal Free Hospital, Pond Street, London, NW3 2QG, UK

**Keywords:** Lipiodol therapy, targeted radiotherapy, primary liver cancer, colorectal liver metastases, in-vitro cytotoxicity

## Abstract

Intra-arterial Lipiodol has been used to deliver targeted therapies to primary, and some metastatic, liver cancers. Targeted radiotherapy has been used by substituting the iodine in Lipiodol with ^131^Iodine (^131^I). Early clinical results are encouraging, but the variable response may partly depend on local pharmacokinetics. This study evaluated the in vitro cytotoxic effects of ^131^I-Lipiodol on human hepatocellular carcinoma (Hep-G2), human colorectal metastatic cancer (SW620), human colorectal hepatic cancer (LoVo) and human umbilical vein endothelial cells (HUVEC) cell lines. The cell cultures were exposed to ^131^I-Lipiodol for 48 h, following which cell counts and viability were assessed by haemocytometer, S-Rhodamine uptake and radioactivity assay. The effect of exposure to control Lipiodol, ^131^I-Lipiodol and ^131^I alone was evaluated. ^131^I-Lipiodol was cytotoxic against all the cancer cell lines but not against the non-malignant (HUVEC) cell line. The cytotoxicity effects were very similar in all the cancer cell lines. There were no cytotoxic effects following exposure to plain ^131^I in any of the cell lines (malignant and non-malignant). A similar trend was seen with radioactivity counts using a gamma counter. The cytotoxic effect of ^131^I-Lipiodol had a graded effect with an increase in cytotoxicity following the increase in the radioactive dose. This study showed that there was a marked cytotoxic effect by ^131^I-Lipiodol on all the cancer cell lines. There was no difference between the controls and the ^131^Iodine. This suggests that effective ^131^I-Lipiodol targeted therapy is dependent on the uptake and retention of Lipiodol by malignant cells. © 1999 Cancer Research Campaign


*British Journal of Cancer* (1999) **79**(11/12), 1665–1671
© 1999 Cancer Research Campaign
Article no. bjoc.1998.0266

### In vitro assessment of Lipiodol-targeted radiotherapy for liver and colorectal cancer cell lines

**RAM Al-Mufti[1], RB Pedley[2], D Marshall[2], RHJ Begent[2], A Hilson[3], MC Winslet[1] and KEF Hobbs[1]**

Departments of [1]Surgery, [2]Clinical Oncology, and [3]Nuclear Medicine, Royal Free Hospital, Pond Street, London NW3 2QG, UK.

**Summary** Intra-arterial Lipiodol has been used to deliver targeted therapies to primary, and some metastatic, liver cancers. Targeted radiotherapy has been used by substituting the iodine in Lipiodol with $^{131}$Iodine ($^{131}$I). Early clinical results are encouraging, but the variable response may partly depend on local pharmacokinetics. This study evaluated the in vitro cytotoxic effects of $^{131}$I-Lipiodol on human hepatocellular carcinoma (Hep-G2), human colorectal metastatic cancer (SW620), human colorectal hepatic cancer (LoVo) and human umbilical vein endothelial cells (HUVEC) cell lines. The cell cultures were exposed to $^{131}$I-Lipiodol for 48 h, following which cell counts and viability were assessed by haemocytometer, S-Rhodamine uptake and radioactivity assay. The effect of exposure to control Lipiodol, $^{131}$I-Lipiodol and $^{131}$I alone was evaluated. $^{131}$I-Lipiodol was cytotoxic against all the cancer cell lines but not against the non-malignant (HUVEC) cell line. The cytotoxicity effects were very similar in all the cancer cell lines. There were no cytotoxic effects following exposure to plain $^{131}$I in any of the cell lines (malignant and non-malignant). A similar trend was seen with radioactivity counts using a gamma counter. The cytotoxic effect of $^{131}$I-Lipiodol had a graded effect with an increase in cytotoxicity following the increase in the radioactive dose. This study showed that there was a marked cytotoxic effect by $^{131}$I-Lipiodol on all the cancer cell lines. There was no difference between the controls and the $^{131}$Iodine. This suggests that effective $^{131}$I-Lipiodol targeted therapy is dependent on the uptake and retention of Lipiodol by malignant cells.

**Keywords:** Lipiodol therapy; targeted radiotherapy; primary liver cancer; colorectal liver metastases; in-vitro cytotoxicity

Lipiodol is an iodinated derivative of poppy seed oil, containing ethyl esters of linoleic, oleic, palmitic and stearic acids, with an iodine content of 38–40% wv (as a naturally iodinated compound) (ABPI, 1991–92; BP, 1993; Chatin and Bonnrmain, 1992). Lipiodol was first shown to be selectively taken up and retained by primary hepatocellular carcinoma in 1979 (Nakakuma et al, 1979), and hepatic metastases of colonic and neuroendocrine tumours in 1988 (Bretagne et al, 1988). This phenomenon of uptake and retention has been used to deliver targeted therapies via the hepatic artery to these tumours (Konno et al, 1983; Nakakuma et al, 1983; Bretagne et al, 1988), either conjugating Lipiodol to cytotoxic agents (such as Epirubicin) to give targeted chemotherapy, or by radiolabelling some of the iodine in Lipiodol with $^{131}$I to deliver targeted radiotherapy (Konno et al, 1983; Bretagne et al, 1988; Novell et al, 1991; Vetter et al, 1991; Al-Mufti et al, 1995). However, the results of clinical trials of its therapeutic value have been unpredictable and rather disappointing (Friedman, 1983; Madden et al, 1993). Several reasons have been given for such poor results, including the lack of in vitro studies of the stability and efficacy of such targeted therapies prior to their clinical use.

Our group have previously reported the in vitro selective uptake and retention of Lipiodol by primary and metastatic hepatic malignancy (Al-Mufti et al, 1995). In this study, an in-vitro assessment of $^{131}$I-Lipiodol was made by studying its cytotoxicity against primary liver and colorectal metastatic cancer cell lines, to investigate the mechanism of action and the therapeutic effect of Lipiodol-targeted therapy.

From our previous studies, in vitro uptake of Lipiodol is at its maximum at 24 h after exposure, and the mean cell cultures doubling time is 19 h (range 18–22 h). Accordingly, the in vitro cytotoxicity in this study was assessed over a period of 48 h.

*Received 1 September 1997*
*Revised 18 May 1998*
*Accepted 7 August 1998*

*Correspondence to:* R A M Al-Mufti

### MATERIALS AND METHODS

### Cell cultures

The malignant cell lines used were Hep-G2 (human hepatoma), LoVo (human colorectal hepatic cancer) and SW620 (human colorectal metastatic cancer). Control non-malignant cells were HUVEC (human umbilical vein endothelial cells). Other non-malignant control cell lines were not readily available for assessment. The cancer cell lines were obtained from the European Collection of Animal Cell Cultures, Porton Down, Salisbury, Wiltshire SP4 0JG, UK.

#### *The culture medium for Hep-G2*

The standard medium recommended by the source laboratory was Dulbecco's modified Eagle's medium (DMEM) with 10% fetal calf serum (FCS), L-glutamine 2 mM $l^{-1}$, penicillin 100 000 units l and streptomycin 100 mg $l^{-1}$; pH at 7.

#### *The culture medium for LoVo*

The standard medium recommended by the source laboratory was Hams F12 or DMEM with 10% FCS, L-glutamine 2 mM $l^{-1}$, penicillin 100 000 units $l^{-1}$ and streptomycin 100 mg $l^{-1}$.

*The culture medium for SW620*

The standard medium recommended by the source laboratory was Leibovitz L15 medium with 10% FCS, L-glutamine 2 mM $l^{-1}$, penicillin 100 000 units $l^{-1}$ and streptomycin 100 mg $l^{-1}$.

*ECCM-culture medium for HUVEC*

M199 medium, 20% FCS, $NaHCO_3$, glutamine, heparin, penicillin and streptomycin; pH 7.2 to 7.3; filtered[17].

### Method for culturing the cancer cell lines

The cancer cell lines (Hep-G2, SW620 and LoVo) were supplied in a cryotube frozen in liquid nitrogen. The cryotube was left at room temperature for 1 min, then transferred to 37°C waterbath for 2 min. The cells in the cryotube were thawed, decanted into a sterile universal with 10 ml of medium (at 4°C) and centrifuged at 90 ***g*** for 7 min. The cell pellet was re-suspended with pre-warmed culture medium (Dulbecco's modified Eagle's medium, DMEM), cultured in a vented 25 $mm^2$ flask and incubated with 5% carbon dioxide humid atmosphere at 37°C. At 90% confluence, the cells were subcultured by discarding the old medium, washing with phosphate-buffered saline (PBS) less $Ca^{2+}/Mg^{2+}$ and then trypsinizing the monolayer with trypsin–EDTA (× 1), for 5–10 min, until the cells were detached. The cells were then suspended in culture medium, centrifuged at 90 ***g*** for 5 min and then subcultured in 96-well plates, seeded at $1 \times 10^4$ cells per well, and incubated overnight at 37°C at 5% carbon dioxide ($CO_2$) and humidity. The cultures were examined 24 h before starting the radioisotope experiment (Leibovitz et al, 1976; Drewinko et al, 1976; Knowles et al, 1980). The mean cell culture doubling times for Hep-G2 and LoVo was 19 h (range 18–20) and for SW620 was 20 h (range 19–21). Full aseptic precautions were used with the cell cultures.

### Method for culturing HUVEC

A fresh umbilical cord was collected from the labour ward in a sterile container with 200 ml of M199 medium and 10% NBCS (new born calf serum). Ethical approval from the Obstetric Unit was obtained. The cord was examined for any damage or clamp marks, selecting a suitable 20 cm segment for culture. The two ends of the umbilical vein were cannulated with a size 16 or 18 F tube and the tubes were secured with silk ties. The vein was flushed with PBS solution (less $Ca^{2+}/Mg^{2+}$), to remove any blood clots, and to detect any leaks. Collagenase 0.1% solution (15 ml) was then injected into the vein and it was incubated at 37°C for 5 min. The collagenase solution was then decanted into a sterile container. An equal volume of M199–20% NBCS was added to neutralize the collagenase, and the suspended cells were centrifuged at 300 ***g*** for 5 min. The cells in the pellet were re-suspended with endothelial cell culture medium (ECCM), cultured in vented flasks (25 $mm^2$) and incubated at 37°C with 5% $CO_2$ humid atmosphere. At 24 h the cultures were washed with 5 ml of M199 medium to remove dead cells and red blood cells, and then the cells were fed with fresh ECCM every 48 h and incubated at 37°C with 5% $CO_2$ and humidity.

When a 90% confluent cell monolayer was reached, the cells were subcultured by discarding the old medium, washing the monolayer with PBS (less $Ca^{2+}/Mg^{2+}$) and then trypsinizing the culture with trypsin-EDTA (× 1) for 3–5 min at 37°C, until cells were detached. The enzyme was then partially neutralized by the addition of 20% NBCS in M199 medium, and the cell suspension was centrifuged at 300 ***g*** for 5 min. Cell counts and viability were determined by trypan blue exclusion using a haemocytometer. The cells in the pellet were resuspended in 100 µl of ECGS (endothelial cell growth supplement) and culture medium (ECCM). The cells were subcultured in 96-well plates, seeded at $1 \times 10^4$ per well and incubated at 37°C with 5% $CO_2$ and humidity, and left overnight to settle. The cultures were examined 24 h before starting the radioisotope experiment (Jaffe et al, 1973; Jaffe, 1987). The mean cell culture doubling time for HUVEC was 20.5 h (range 19–22).

### Radioisotopes

$^{131}$I-Lipiodol used in this study was supplied by CIS in France (via CIS [UK] Limited, Dodding House, Wellington Road, High Wycombe HP12 3PR, UK), which was identical to the $^{131}$I-Lipiodol used therapeutically. The radioactive dose of Iodine 131 ($^{131}$I) used was according to a dose-titration method as shown below.

### General method

Each of the cell lines were subcultured in 96-well plates; cells were seeded at $1 \times 10^4$ cells per well. The cell cultures were allowed to grow overnight in a humidified incubator with an atmosphere of 5% $CO_2$ at 37°C. At 24 h (zero hour), the old medium was discarded and replaced with control or radioisotope culture medium according to the appropriate concentration and radioactivity, and the cultures were then allowed to grow for a further period of up to 48 h. Lipiodol was emulsified with diatrizoate (urografin) into an aqueous-based emulsion, similar to the standard clinical use of urografin prior to the arterial administration of Lipiodol. The cell cultures were treated as follows:

1. 100 µl medium in each of the control wells (first control), with culture medium only.
2. 100 µl cold-Lipiodol medium (4%) in each of the Lipiodol control wells (second control).
3. 100 µl of plain $^{131}$I 40 µCi $ml^{-1}$ in each of the radioactive control wells (third control).
4. 100 µl of the $^{131}$I-Lipiodol 10 µCi $ml^{-1}$ emulsion in each of the $I^{131}$ low-dose wells (equivalent to 4% Lipiodol).
5. 100 µl of the $^{131}$I-Lipiodol 20 µCi $ml^{-1}$ emulsion in each of the $I^{131}$ medium-dose wells (= 4% Lipiodol). This is equivalent to the radio-therapeutic clinical dose.
6. 100 µl of the $^{131}$I-Lipiodol 40 µCi $ml^{-1}$ emulsion in each of the $I^{131}$ high-dose wells (equivalent to 4% Lipiodol).

The cultures were incubated at 37°C with 5% $CO_2$ for 48 h in a special shielded (radioisotope) room under full radiation protection control. The experiment was repeated three times with four sets of plates for each cell line, each containing six identical wells for each test dose of radioactivity. At the end of the incubation period, the control and radioactive culture media were discarded appropriately and the cell monolayers were washed with PBS solution three times. One set of the plates was then processed for cell density and viability using the sulphorhodamine B (SRB) assay (Skehan et al, 1990). Another set of the plates was processed for measurement of cellular radioactivity using a gamma scintillation counter (Pharmacia, 1470 Wizard). The 96-well plates were

 © Cancer Research Campaign 1999

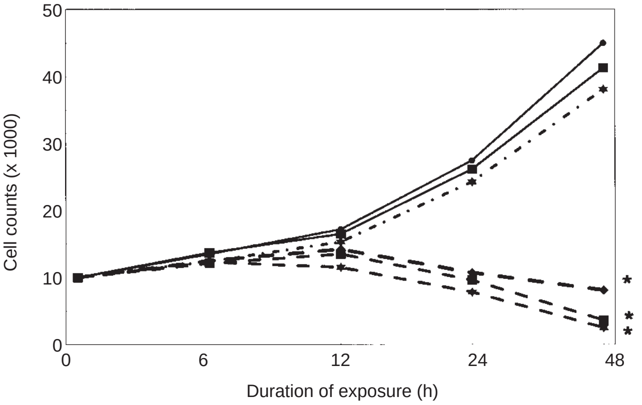

**Figure 1** Effects of $^{131}$I-Lipiodol on the cell growth of Hep-G2 hepatoma cell line, compared to control Lipiodol and control $^{131}$I. Similar effects were seen in other cancer cell lines. *$P < 0.0001$ compared to controls. Control, — ● —; Lipiodol control, — ■ —; Control $^{131}$I (40 μCi ml$^{-1}$), – .✰. –; $^{131}$I-Lipiodol (10 μCi ml$^{-1}$), – ◆ –; $^{131}$I-Lipiodol (20 μCi ml$^{-1}$), – ■ –; $^{131}$I-Lipiodol (40 μCi ml$^{-1}$), – ✰ –

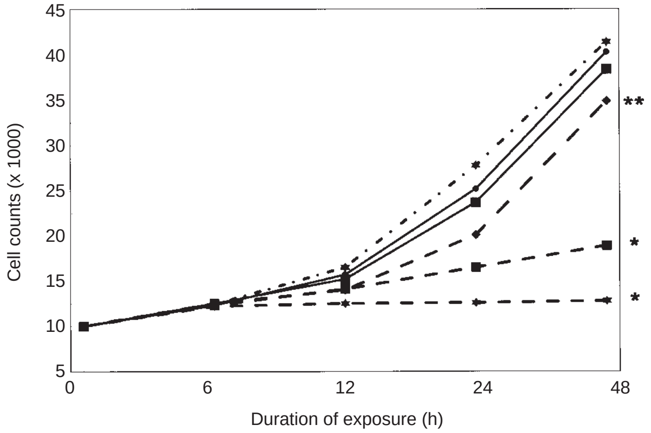

**Figure 2** Effects of $^{131}$I-Lipiodol on the cell growth of non-malignant endothelial cells (HUVEC). *$P < 0.00001$, **$P = 0.04$ compared to controls. Control, – ● –; Lipiodol control, — ■ —; Control $^{131}$I (40 μCi ml$^{-1}$), – .✰. –; $^{131}$I-Lipiodol (10 μCi ml$^{-1}$), – ◆ –; $^{131}$I-Lipiodol (20 μCi ml$^{-1}$), — ■ —; $^{131}$I-Lipiodol (40 μCi ml$^{-1}$), – .✰. –

put into the gamma scintillation counter using the appropriately selected mode of radioactivity for $^{131}$I after calibration, and a computerized readout was obtained. Cell viability and counts were further assessed with the standard trypan blue exclusion method using the haemocytometer. After trypsinization, the cells were suspended in medium, and the cell count was calculated for each well. Nine readings were calculated per well and the mean value was obtained. The total cell count was calculated by multiplying the mean result by the total volume in which the cells were suspended. Light and electron microscopy examinations were also used to assess cytotoxicity and uptake of radioactivity.

### Method for the sulphorhodamine B (SRB) test

This test was used to measure cell density after treatment, by using protein analysis. At the end of the experiment (exposure to

**Table 1** Effects of $^{131}$I-Lipiodol on Hep-G2 (human hepatoma) at 48 h

| **Hep-G2 cell line** *n* **= 6 (s.d.)** | **Mean d.p.m. counts (gamma counter)** | **Mean cell counts (× 10$^3$) (haemocytometer)** | **Viable cells (trypan blue) (%)** |
|---|---|---|---|
| Control wells | 191.9 (21.3) | 45 (3.1) | 98.1 |
| 4% Lipiodol control wells | 211.7 (34.5) | 41.3 (7.1) | 97.6 |
| Plain I$^{131}$ (40 μCi ml$^{-1}$) | 365.2 (26.7) | 38.1 (3.8) | 97.8 |
| Low-dose $^{131}$I-Lipiodol (10 μCi ml$^{-1}$) | 2566.3 (51.3) | $^a$8.1 (2.7) | *4.7 |
| Medium-dose $^{131}$I-Lipiodol (20 μCi ml$^{-1}$) | 5565.9 (44.8) | $^a$5.6 (1.8) | *3.2 |
| High-dose $^{131}$I-Lipiodol (40 μCi ml$^{-1}$) | 17 362.1 (47.2) | $^a$4 (1.5) | *1.9 |

*n* = 6; d.p.m. = disintegration per min; standard deviation in parentheses. Number of cells at the start of experiment was 10 × 10$^3$ cells per well. $^a$*P*-value = < 0.0001.

**Table 2** Effects of $^{131}$I-Lipiodol on LoVo (human colorectal hepatic cancer) at 48 h

| **LoVo cell line** *n* **= 6 (s.d.)** | **Mean d.p.m. counts (gamma counter)** | **Mean cell counts (× 10$^3$) (haemocytometer)** | **Viable cells (trypan blue) (%)** |
|---|---|---|---|
| Control wells | 195.7 (29.1) | 57.5 (5.8) | 97.2 |
| 4% Lipiodol control wells | 182.7 (41.8) | 53.1 (8.2) | 97.5 |
| Plain I$^{131}$ (40 μCi ml$^{-1}$) | 591.1 (30.3) | 55.6 (6.3) | 98.1 |
| Low-dose $^{131}$I-Lipiodol (10 μCi ml$^{-1}$) | 6317.6 (44.9) | $^a$16.3 (3.4) | $^a$6.0 |
| Medium-dose $^{131}$I-Lipiodol (20 μCi ml$^{-1}$) | 13 421.2 (46.2) | $^a$12.1 (2.9) | $^a$4.2 |
| High-dose $^{131}$I-Lipiodol (40 μCi ml$^{-1}$) | 22 161.3 (41.5) | $^a$7.1 (2.3) | $^a$2.4 |

*n* = 6; d.p.m. = disintegration per minute; standard deviation in parentheses. Number of cells at the start of experiment was 10 × 10$^3$ cells per well. $^a$*P*-value = < 0.001.

*© Cancer Research Campaign 1999*

**Table 3** Effects of $^{131}$I-Lipiodol on SW620 (human colorectal metastatic cancer) (48 h)

| SW20 cell line *n* = 6 (s.d.) | Mean d.p.m. counts (gamma counter) | Mean cell counts (× 10³) (haemocytometer) | Viable cells (trypan blue) (%) |
|---|---|---|---|
| Control wells | 176.4 (20.8) | 48.8 (3.2) | 96.2 |
| 4% Lipiodol control wells | 164.5 (32.1) | 48.1 (5.4) | 95.3 |
| Plain $I^{131}$ (40 μCi $ml^{-1}$) | 266.4 (37.4) | 52.5 (3.9) | 97.1 |
| Low-dose $^{131}$I-Lipiodol (10 μCi $ml^{-1}$) | 5334.8 (46.3) | $^{a}$15.6 (2.8) | $^{a}$5.2 |
| Medium-dose $^{131}$I-Lipiodol (20 μCi $ml^{-1}$) | 14 973.1 (39.7) | $^{a}$8.9 (3.3) | $^{a}$2.8 |
| High-dose $^{131}$I-Lipiodol (40 μCi $ml^{-1}$) | 26 712.1 (43.2) | $^{a}$3.9 (1.1) | $^{a}$1.4 |

*n* = 6; d.p.m. = disintegration per minute; standard deviation in parentheses. Number of cells at the start of experiment was 10 × 10³ cells per well. $^{a}$*P*-value = < 0.0001.

**Table 4** Effects of $^{131}$I-Lipiodol on HUVEC (human umbilical vein endothelial cells) (48 h)

| HUVEC cell line *n* = 6 (s.d.) | Mean d.p.m. counts (gamma counter) | Mean cell counts (× 10³) (haemocytometer) | Viable cells (trypan blue) (%) |
|---|---|---|---|
| Control wells | 144.2 (39.2) | 40.3 (3.4) | 95.8 |
| 4% Lipiodol control wells | 176.4 (45.7) | 39.4 (6.2) | 97.1 |
| Plain $I^{131}$ (40 μCi $ml^{-1}$) | 375.9 (52.2) | 41.4 (4.7) | 98.2 |
| Low-dose $^{131}$I-Lipiodol (10 μCi $ml^{-1}$) | 8844.9 (48.3) | $^{b}$35.9 (3.1) | $^{b}$85.4 |
| Medium-dose $^{131}$I-Lipiodol (20 μCi $ml^{-1}$) | 9202.2 (53.2) | $^{a}$18.9 (3.2) | $^{b}$84.7 |
| High-dose $^{131}$I-Lipiodol (40 μCi $ml^{-1}$) | 9023.1 (46.8) | $^{a}$12.8 (2.5) | $^{b}$80.4 |

*n* = 6; d.p.m. = disintegration per minute; standard deviation in parentheses. Number of cells at the start of experiment was 10 × 10³ cells per well, $^{a}$ *P*-value = < 0.0001, $^{b}$*P*-value = 0.04.

radioisotopes or controls), the medium was discarded and the wells were rinsed with PBS and the cells were then exposed to 100 μl of cold 10% wv trichloroacetic acid for 1 h at 4°C. The plates were then washed with water and air dried. The SRB assay was commenced by dissolving 0.4% wv SRB in 1% acetic acid and adding 100 μl to each well to stain the fixed cells for 30 min. The dye was then removed and immediately rinsed with 1% acetic acid four times and then air dried. The assay reading was commenced by solubilizing the bound dye with 100 μl 10 mM (0.121 g 100 $ml^{-1}$) Tris base, pH 10.5, for 15 min on a gyratory shaker. The cell density was calculated for each culture using a 96-well plate reader at 490 nm (Skehan et al, 1990).

### RESULTS

Using previously described method, Lipiodol uptake by all the cancer cell lines and by the endothelial cells was demonstrated on light and electron microscopy as early as 3 h (Al-Mufti et al, 1996). In this study, $^{131}$I-Lipiodol was also demonstrated within the cancers cells and the benign endothelial cells as early as 6 h. Lipiodol alone as a control (without $^{131}$I) had no effect on the cell growth or viability when compared to the controls, and was therefore not cytotoxic to any of the cell lines used in this study. At early stages of exposure, there were no cytotoxic effects seen as expected.

$^{131}$I-Lipiodol was found to be highly cytotoxic against all the three malignant cell lines (Hep-G2, LoVo and SW620) at the low-, medium- and high-dose range ($P$ = 0.0001, Figure 1). The cytotoxicity effects of $^{131}$I-Lipiodol increased with increasing doses of radioactivity, with optimal toxicity being achieved with the high-dose (40 μCi $ml^{-1}$). The results are summarized in Tables 1–4. The effects of $^{131}$I-Lipiodol on Hep-G2, LoVo and SW620 were identical, and the cytotoxicity was maximal at 48 h of exposure. $^{131}$I-Lipiodol was not cytotoxic to endothelial cells (Figure 2, Table 4) but did reduce the rate of cell growth (cytostatic effect). The percentage of endothelial cell viability remained high (87–93%) after exposure to the three different doses of $^{131}$I-Lipiodol, compared to 96% cell viability in the control cultures.

$^{131}$I alone did not have any cytotoxic effect against any of the cell lines used (malignant and non-malignant cells), even at high dosage (40 μCi $ml^{-1}$) despite the cells being in direct contact with the radioiodine (Figures 1 and 2). In fact the cell growth and count of the control endothelial cells was slightly higher after exposure to $^{131}$I alone, and all the cultures (malignant and non-malignant) maintained a high percentage of cell viability (97–98%).

Light and electron microscopy confirmed the uptake of Lipiodol in the form of cytoplasmic membrane-bound vesicles in all the cell lines (malignant and non-malignant) as illustrated in Figure 3A. Electron microscopy also confirmed the uptake of $^{131}$I-Lipiodol by

 *© Cancer Research Campaign 1999*

**Table 5** The sulphorhodamine B (SRB) test for cell density assay (48 h)

| Cell lines | Control | Cell density measurements using the sulforhodamine B assay | | | | |
|---|---|---|---|---|---|---|
| | | Lipiodol control | $^{131}$I control | $^{131}$I-Lipiodol 10 $\mu$Ci ml$^{-1}$ | $^{131}$I-Lipiodol 20 $\mu$Ci ml$^{-1}$ | $^{131}$I-Lipiodol 40 $\mu$Ci ml$^{-1}$ |
| Hep-G2 | 0.074 | 0.071 | 0.069 | 0.013* | 0.010* | 0.002* |
| LoVo | 0.049 | 0.054 | 0.048 | 0.015* | 0.012* | 0.006* |
| SW620 | 0.039 | 0.036 | 0.037 | 0.007* | 0.003* | 0.002* |
| HUVEC | 0.044 | 0.039 | 0.049 | 0.036 | 0.042 | 0.050 |

$n$ = 6; results are the mean absolute absorbance values for the cell density index. $^{a}P$-value = < 0.001.

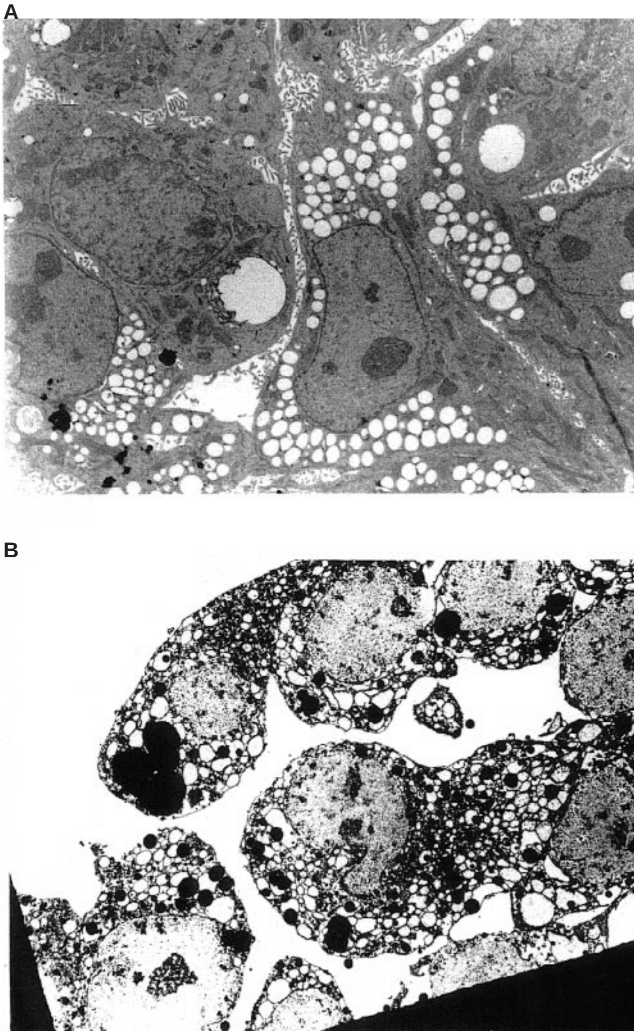

**Figure 3** Electron micrographs of Hep-G2 cancer cells. (**A**) Healthy cells with cytoplasmic Lipiodol in membrane-bound vesicles, following exposure to control Lipiodol. (**B**) $^{131}$I-Lipiodol particles inside the dead cancer cells, following exposure to $^{131}$I-Lipiodol

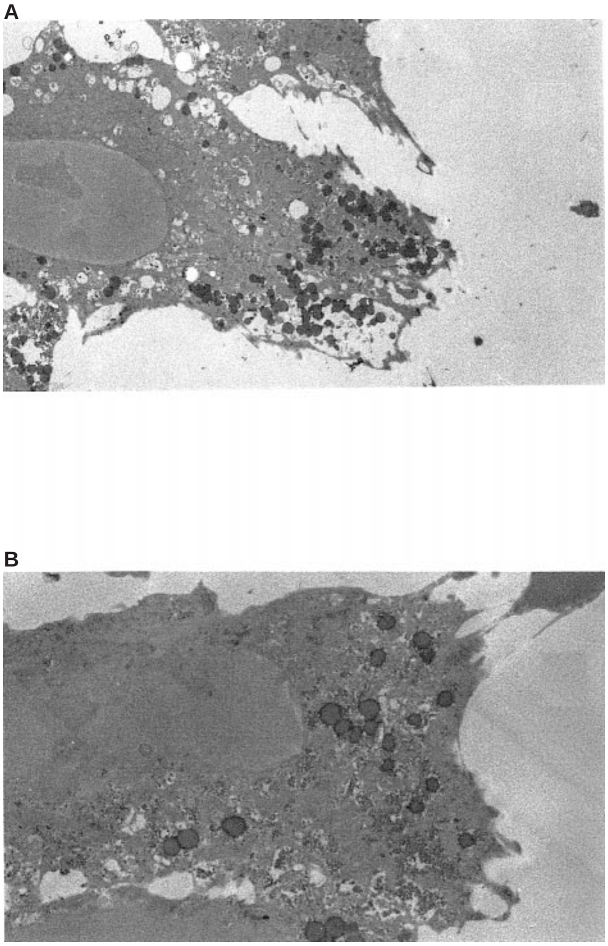

**Figure 4** Electron micrographs of HUVEC endothelial cells. (**A**) Healthy cells with cytoplasmic membrane-bound vesicles of lipid, following exposure to control Lipiodol. (**B**) $^{131}$I-Lipiodol particles inside the healthy benign cells, following exposure to $^{131}$I-Lipiodol all the cells and the cytotoxic effects of $^{131}$I-Lipiodol on the cancer cell lines (Figure 3B). There was 100% cell death at 48 h, leaving cell shadows and cytoskeletons of dead cancer cells still containing $^{131}$I-Lipiodol vesicles (Figure 3B). In contrast, electron microscopic study of the non-malignant control endothelial cells confirmed the high percentage of cell viability (percentage of viable cells 80–85%) at 48 h of exposure to $^{131}$I-Lipiodol, in spite of its uptake by the endothelial cells, as illustrated in Figure 4.

Sulphorhodamine B test and gamma scintillation counting (Table 5) correlated well with the cell growth curves, cell viability assays and electron microscopy. All the cell lines showed high cellular radioactivity following exposure to $^{131}$I-Lipiodol, but the

*© Cancer Research Campaign 1999*

radioactivity remained low following exposure to $^{131}$I alone, without Lipiodol (Tables 1–4). This confirmed that Lipiodol was essential for transporting $^{131}$I into the cells to achieve its preferential cytotoxicity effects in the malignant cells (i.e. intracellular radiotherapy).

### DISCUSSION

This study confirms that the uptake of Lipiodol is not specific to cancer cell lines, as the non-malignant endothelial cells take up Lipiodol as well. However, $^{131}$I-Lipiodol is selectively and highly cytotoxic against all the cancer cell lines but not against the benign endothelial cells. Although the mechanism of Lipiodol uptake is poorly understood, this study confirms our previous reports that Lipiodol is taken up and is retained in the form of cytoplasmic membrane-bound vesicles (Al-Mufti et al, 1995). Quantification methods using computer-assisted image analysis of the uptake of Lipiodol by the malignant and the non-malignant cell lines (Al-Mufti et al, 1996) have revealed that cancer cell lines, unlike non-malignant cells, are unable to expel Lipiodol. This may further enhance the cytotoxic effect of $^{131}$I-Lipiodol in cancer cells.

Lipiodol alone does not appear to have any cytotoxic effect against any of the cell lines studied. This confirms the clinical observations seen in many in vivo studies of Lipiodol-targeted therapies of liver cancers. It is noteworthy that, in this study, $^{131}$I alone without the Lipiodol has no cytotoxic effect against any of the cell lines tested. This is in spite of the cell cultures being exposed directly to a high and comparable dose of $^{131}$I radioactivity in their culture medium (extracellular radiation). This is the first study to show the importance of using Lipiodol to deliver $^{131}$I, by virtue of its ability to deliver the isotope inside the cancer cells (intracellular radiotherapy), even if in membrane-bound vesicles. There is no separation of the $^{131}$I from Lipiodol during the process of uptake by the cancer cells, as Lipiodol is a naturally iodinated oil compound, with an iodine content of 38–40%. $^{131}$I-Lipiodol shows cytotoxic effects against the cancer cell lines even when the radioactive dose is very small, a quarter of the radioactivity of the $^{131}$I alone.

The morphological changes observed with light and electron microscopy, following $^{131}$I-Lipiodol administration, confirmed intracellular $^{131}$I-Lipiodol vesicles inducing acute cell death of cancer cells, with maximum effect seen at 24 and 48 h, with little effects at the early stages of exposure (3 and 6 h) as expected. However, the effects on the non-malignant endothelial cells were only cytostatic, giving a reduction in the rate of cell growth, but a high percentage viable cells. This mechanism may operate in vivo when Lipiodol-targeted therapies are used. The long-term apoptotic effects of $^{131}$I-Lipiodol on the control endothelial cells were not evaluated further in this study.

As Lipiodol is a naturally iodized oil with a higher density than the aqueous culture media, one could argue that by leaving the culture media incubated for 48 h inside the incubator, the $^{131}$I-Lipiodol component of the emulsion could then separate and sink into the bottom of the wells and would come into intimate contact with cell monolayers at the base of the wells. However, it is unlikely that this had any significant effect on the results in this study for three reasons. Diatrizoate (urografin) was used to emulsify the Lipiodol (as it is commonly used clinically prior to the arterial administration, which would maintain the $^{131}$I-Lipiodol in its suspended form much longer), together with the use of human endothelial cells as benign control cells and the use of much smaller doses of $^{131}$I-Lipiodol to compare to that of $^{131}$I alone (down to a quarter of the radioactive dose of $^{131}$I). If the $^{131}$I-Lipiodol were to sink down to the bottom of the wells, then one would expect that not only the cancer cells, but all the endothelial cells, to be killed because of the intimate contact with a high dose of radioactivity. There was a significant cytotoxic effect seen with the use of even a low dose of $^{131}$I-Lipiodol (0.01 $\mu$Ci $\mu$l$^{-1}$), compared to a much higher dose of radioactivity with$^{131}$I alone (0.04 $\mu$Ci $\mu$l$^{-1}$), which had no cytotoxic effect.

The use of the radioactive form of Lipiodol as a potentially effective treatment for patients with hepatocellular carcinoma and some colorectal hepatic metastases is supported by the effective localization and retention of Lipiodol by human hepatocellular and colorectal metastatic cancers in vitro. The results of $^{131}$I-Lipiodol cytotoxicity in Hep-G2, LoVo and SW620 in this study indicate that $^{131}$I-Lipiodol is highly cytotoxic to such tumours that show good uptake of Lipiodol, and that the radiation lethal dose can be as low as 0.01 $\mu$Ci $\mu$l$^{-1}$ in cell cultures, with a steep dose–response relationship. Lipiodol-targeted radiotherapy would have a limited effect against tumours that show poor Lipiodol uptake. Hepatocellular carcinoma and metastatic colorectal cancers have been regarded as resistant to radiotherapy, but this study has shown that these tumours may be sensitive to intracellular radiotherapy with $^{131}$I-Lipiodol. This is particularly significant, since highly effective cytotoxic dosages of $^{131}$I-Lipiodol can be administered intra-arterially quite safely in patients with these liver cancers with minimal cytotoxicity to the normal liver (Raoul et al, 1988). The only limiting factor for Lipiodol-targeted radiotherapy, as seen in the clinical trials, is the variability of the in vivo uptake of Lipiodol by cancer cells. This is dependent on the intra-arterial delivery of Lipiodol to the cancer. If the tumour uptake of Lipiodol can be improved, then better cytotoxic effects of Lipiodol-targeted radiotherapy should be achieved. Future clinical studies should be directed towards improving the in vivo uptake of Lipiodol, for example by combining this form of targeted therapy with vasoactive agents such as angiotensin.

In conclusion, in vitro cytotoxicity assay of $^{131}$I-Lipiodol revealed it to be cytotoxic against cells taken from human hepatocellular carcinoma and colorectal metastatic cancers but not to benign endothelial cells. This was related to the selective uptake and retention of Lipiodol by the cancer cell lines, with evidence of intra-cytoplasmic accumulation of $^{131}$I-Lipiodol (intracellular radiation). Equivalent or higher doses of radioactivity delivered to the cells in the absence of Lipiodol (i.e. extracellular radiation) had no toxic effect.

### ACKNOWLEDGEMENTS

The authors acknowledge the financial support from the Basildon and South Essex Medical Education and Research Trust (Basildon, Essex, SS16 5NL, UK), and the Cancer Research Campaign Trust (CRC, London, UK).

The authors also acknowledge the help of Mr John Wood at the Department of Nuclear Medicine, Royal Free Hospital, and of Dr J Bomanji at the Institute of Nuclear Medicine, University College London Hospital, for their help in the handling of $^{131}$I-Lipiodol.

© Cancer Research Campaign 1999

### REFERENCES

ABPI Data Sheet Compendium (1991–1992) *The Pharmaceutical Industry*, compiled by G Walker, pp. 1199. Datapharm; London

Al-Mufti R, Bhattacharya S, Novell JR, Winslet MC and Hobbs KEF (1995*a*) Intra-arterial radiotherapy with Lipiodol-Iodine 131 for irresectable hepatocellular carcinoma. *Br J Cancer* **72**: 19

Al-Mufti R, Bhattacharya S, Lewin J, Winslet MC and Hobbs KEF (1995*b*) In vitro assessment of iodised oil (Lipiodol) uptake in primary and metastatic liver tumours. *Br J Cancer* **72**: 38

Al-Mufti RAM, Bhattacharya S, Winslet MC and Hobbs KEF (1996*a*) Quantification of uptake and retention of iodised oil (Lipiodol) in primary and secondary hepatic malignancy (Abstract presentation). *HPB Surgery* **9**: 72 [P006]

Al-Mufti RAM, Bhattacharya S, Hobbs KEF and Winslet MC (1996*b*) In-vitro assessment of the uptake of Lipiodol by liver tumours: is it a mechanism specific to Lipiodol? (Abstract). *HPB Surgery* **9**: 72 [P005]

Bhattacharya S, Dhillon AP, Winslet MC, Davidson BR, Shukla N, Datta Gupta S, Al-Mufti RAM and Hobbs KEF (1996) Human liver cancer cells and endothelial cells incorporate iodised oil. *Br J Cancer* **73**: 877–881

BP (1993) *The British Pharmacopoeia 1993*, Volume II, pp. 967. Published on the Recommendation of the Medicines Commission Pursuant to the Medicines Act 1968. HMSO: London

Bretagne J-F, Raoul J-L, Bourget P, Duvauferrier R, Deugneir Y, Faroux R, Ramée A, Herry J-Y and Gastard J (1988) Hepatic artery injection of I-131 labelled lipiodol part II: preliminary results of therapeutic use in patients with hepatocellular carcinoma and liver metastases. *Radiology* **168**: 547–550

Chatin I and Bonnrmain B (1992) *Composition de Lipiodol Ultra Fluide*. Laboratoire Guerbet: France

Chou FI, Lui WY, Chi CW and Chan WK (1994) $^{131}$I-Lipiodol cytotoxicity in hepatoma cells. Proceedings of the National Science Council, ROC, Part B: *Life Sciences* **18**: 154–160

Drewinko B, Romsdahl MM, Yang LY, Ahearn MJ and Trujillo JM (1976) Establishment of a human carcinoembryonic antigen-producing colon adenocarcinoma cell line. **36**: 467–475

Friedman MA (1983) Primary hepatocellular cancer: present results and future prospects. *Int J Radiat Oncol Biol Phys* **9**: 1841–1850

Jaffe EA (1987) Cell biology of endothelial cells. *Hum Pathol* **18**: 234–239

Jaffe EA, Nachman RL and Becker CG (1973) Culture of human endothelial cells derived from umbilical veins. Identification by morphologic and immunologic criteria. *J Clin Invest* **52**: 2745–2756

Knowles BB, Howe CC and Aden DP (1980) Human hepatocellular carcinoma cell lines secrete the major plasma proteins and hepatitis B surface antigen. *Science* **209**: 497–499

Konno T, Maeda H and Iwai K (1983) Effect of arterial administration of high molecular weight anticancer agent SMANCS with lipid lymphographic agent on hepatoma. *Eur J Cancer Clin Oncol* **19**: 1053–1965

Konno T, Maeda H, Iwai K, Maki S, Tashiro S, Uchida M and Miyauchi Y (1984) Selective targeting of anti-cancer drug and simultaneous image enhancement in solid tumours by arterially administered lipid contrast medium. *Cancer* **54**: 2367–2374

Leibovitz A, Stinson JC, McCombs WB, McCoy CE, Mazur KC and Mabry ND (1976) Classification of human colorectal adenocarcinoma cell line. *Cancer Res* **36**: 4562–4569

Madden MV, Krige JEJ, Bailey S, Beningfield SJ, Geddes C, Werner ID and Terblanche J (1993) Randomised trial of targeted chemotherapy with lipiodol and 5-epidoxorubicin compared with symptomatic treatment for hepatoma. *Gut* **34**: 1598–1600

Nakakuma K, Tashiro S and Uemura K (1979) An attempt for increasing effects of hepatic artery ligation for advanced hepatoma (English abstract). *Jap-Deutsche Med Beriehte* **24**: 675–682

Nakakuma K, Tashiro S, Hiraoka T, Uemura K, Konno T, Miyauchi Y and Yokoyama I (1983) Studies on anticancer treatment with an oily anticancer drug injected into the ligated feeding hepatic artery for liver cancer. *Cancer* **52**: 2193–2200

Novell R, Hilson A and Hobbs K (1991) Ablation of recurrent primary liver cancer using $^{131}$I-Lipiodol. *Postgrad Med J* **67**: 393–395

Raoul J-L, Bourguet P, Bretagne JF, Duvanferrier R, Coornaet S, Darnault P, Rammée, Herry JY and Gastard J (1988) Hepatic artery injection of 131-I-labelled lipiodol. Part I: Biodistribution study results in patients with hepatocellular carcinoma and liver metastases. *Radiology* **168**: 541–545

Skehan P, Storeng R, Scudiero D, Monks A, McMahon J, Vistica D, Warren JT, Bokesch H, Kenney S and Boyd MR (1990) New colorimetric cytotoxicity assay for anticancer-drug screening. *J Natl Cancer Inst* **82**: 1107–1112

Vetter D, Wenger J-J, Bergier J-M, Doffoel M and Bokel R (1991) Transcatheter oily chemoembolization in the management of advanced hepatocellular carcinoma in cirrhosis: results of a Western comparative study in 60 patients. *Hepatology* **13**: 427–433

*© Cancer Research Campaign 1999*
